# Optoelectronic Evaluation and Loss Analysis of PEDOT:PSS/Si Hybrid Heterojunction Solar Cells

**DOI:** 10.1186/s11671-016-1790-1

**Published:** 2017-01-09

**Authors:** Zhenhai Yang, Zebo Fang, Jiang Sheng, Zhaoheng Ling, Zhaolang Liu, Juye Zhu, Pingqi Gao, Jichun Ye

**Affiliations:** 1 Ningbo Institute of Material Technology and Engineering, Chinese Academy of Sciences, Ningbo, 315201 China; 2 Department of Physics, Shaoxing University, Shaoxing, 312000 China

**Keywords:** Hybrid solar cells, Optoelectronic loss, PEDOT:PSS/Si, 85.60.-q, Optoelectronic device, 84.60.Jt, Photovoltaic conversion

## Abstract

The organic/silicon (Si) hybrid heterojunction solar cells (HHSCs) have attracted considerable attention due to their potential advantages in high efficiency and low cost. However, as a newly arisen photovoltaic device, its current efficiency is still much worse than commercially available Si solar cells. Therefore, a comprehensive and systematical optoelectronic evaluation and loss analysis on this HHSC is therefore highly necessary to fully explore its efficiency potential. Here, a thoroughly optoelectronic simulation is provided on a typical planar polymer poly (3,4-ethylenedioxy thiophene):polystyrenesulfonate (PEDOT:PSS)/Si HHSC. The calculated spectra of reflection and external quantum efficiency (EQE) match well with the experimental results in a full-wavelength range. The losses in current density, which are contributed by both optical losses (i.e., reflection, electrode shield, and parasitic absorption) and electrical recombination (i.e., the bulk and surface recombination), are predicted via carefully addressing the electromagnetic and carrier-transport processes. In addition, the effects of Si doping concentrations and rear surface recombination velocities on the device performance are fully investigated. The results drawn in this study are beneficial to the guidance of designing high-performance PEDOT:PSS/Si HHSCs.

## Background

Although conventional *p-n* junction silicon solar cells (SCs) dominate photovoltaic (PV) market, the relevant applications have been substantially restricted by relatively high production cost, which can be partially attributed to their complicated fabrication process [1]. Recently, organic/silicon (Si) hybrid heterojunction solar cells (HHSCs) that combine the advantages of the Si base with the organic functional layer have attracted much attention [2, 3]. In particular, a *p*-type polymer of poly(3,4-ethylenedioxy thiophene):polystyrenesulfonate (PEDOT:PSS) with a relatively high work function and a wide bandgap has been widely used in HHSCs as a hole-conductive material [4–7]. According to previous reports, power conversion efficiencies (PCEs) of over 13% have been achieved for PEDOT:PSS/Si HHSCs by a simple spin-coating method, demonstrating their great potentials in future photovoltaic application [8–16].

However, compared to the traditional SCs, the relatively poor PCE for this kind of HHSC is still the main challenge that prevent them from becoming a competitive PV technology. Chi et al. demonstrated that the conductivity and wettability of the PEDOT:PSS film can be markedly improved by incorporating different additives into the PEDOT:PSS solution, and the performance of PEDOT:PSS/Si HHSCs was greatly enhanced accordingly [17]. Yu et al. reported a PCE of up to 13.7% for PEDOT:PSS/Si HHSCs on nanostructured Si through engineering the interface by adding a solution-processed cesium carbonate layer [18]. Liu et al demonstrated a PCE of 15.5% due to increased conductivity through the addition of *p*-toluenesulfonic acid into PEDOT:PSS as well as enhanced light-harvesting capabilities by employing an antireflection layer of TiO_2_ [19]. Despite the routine increases in PCE of PEDOT:PSS/Si HHSCs, the cognition of researchers for such HHSCs has not yet reached a level of omnidirectional management. Specially, a qualitative analysis combining a thoroughly optoelectronic evaluation and the recombination mechanism for PEDOT:PSS/Si HHSCs is still lacking, which heavily limits the further design and construction of high-efficiency PEDOT:PSS/Si HHSCs.

In this paper, we focus particularly on the optoelectronic properties of planar PEDOT:PSS/Si HHSCs. We reproduce the optical and the electrical performance of our experimental results by accurate numerical simulation. In addition, we also present an extended loss analysis for this kind of devices by addressing the optical absorption/reflection properties and carrier transport/recombination process inside the HHSCs. The optical losses including top shielding loss by electrode, parasitic absorption in PEDOT:PSS, and rear metal electrode, as well as reflection by the front interface, are lumped. The bulk and surface recombination that affect the external quantum efficiency (EQE) of the HHSCs are also described. Moreover, to comprehensively track the loss mechanism, the optoelectronic responses of PEDOT:PSS/Si HHSCs under different doping concentrations of Si substrate and surface recombination velocities are also simulated.

## Methods

**Fig. 1:**
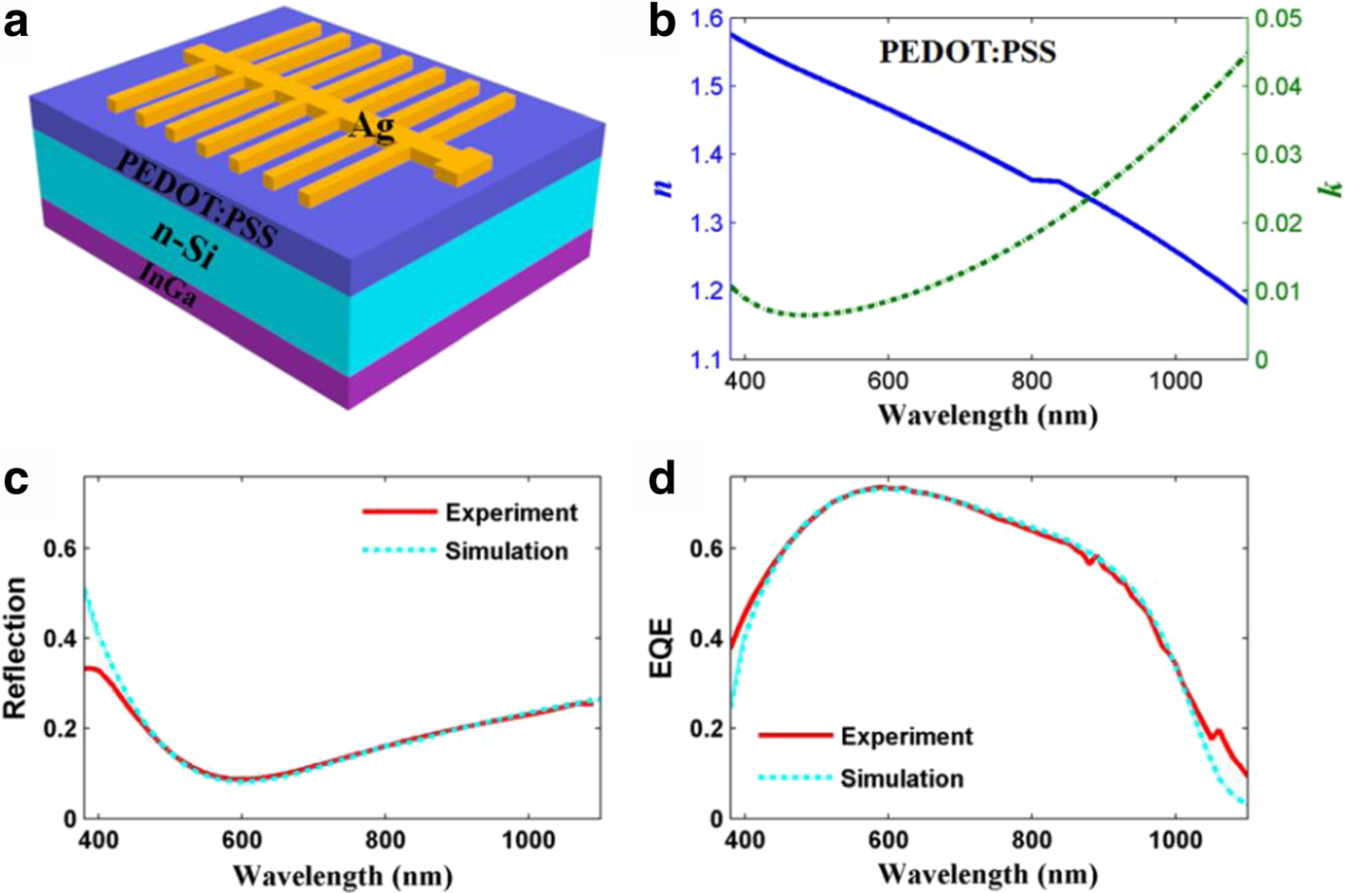
**a** Simulated device of Ag-grid/PEDOT:PSS/*n*-Si/InGa configuration. **b** Refractive index of PEDOT:PSS used in this study. **c**, **d** The simulated and measured reflection/EQE spectrum of the HHSCs

Experimental and simulated configuration of the planar PEDOT:PSS/Si HHSCs was briefly depicted in Fig. 1a, where silver (Ag) and indium-gallium (InGa) were employed as front and rear electrodes, respectively. The *n*-type-doped Si with a thickness of 300 μm and a resistivity of 1~5 Ω·cm (i.e., doping concentration, 1.0~4.7 × 10^15^ cm^–3^) was used in our experiment, which is well matched with *p*-type PEDOT:PSS. Detailed experimental fabrication process can be found in our previous publications [6, 8, 13, 16]. A highly conductive PEDOT:PSS with thickness of ~103 nm was spin coated on the front surface of Si to work as an antireflection and hole-conductive layer [20], as well as to form a junction [21]. In this research, we regarded the PEDOT:PSS/Si contact as a *p-n* heterojunction, because the strong inversion layer that formed in the Si and PEDOT:PSS interface can effectively separate electron-hole pairs and the relative high potential barrier prevents the electron from diffusing into the PEDOT:PSS layer [22].

In order to evaluate the device performance in both optical and electrical domains, we performed photoelectrical simulation under the platform of COMSOL Multiphysics, which is based on finite element method (FEM) [23]. By solving the Maxwell’s equations, we predicted the optical characteristics of HHSCs, including light absorption and reflection. The electrical responses including carrier generation, transportation, recombination, and collection were obtained by imitating the detailed carrier behaviors inside the HHSCs. In this way, the reflection of the entire system (as shown in Fig. 1c) and the EQE of the HHSCs (as shown in Fig. 1d) can be obtained easily. Moreover, the optical constant (i.e., reflective index (*n*) and extinction coefficient (*k*)) of PEDOT:PSS was measured by a J. A. WoollamM-2000DI the spectroscopic ellipsometry, as plotted in Fig. 1b. The optical parameters of the other materials are taken from Palik’s data [24].

## Results and Discussion

**Fig. 2:**
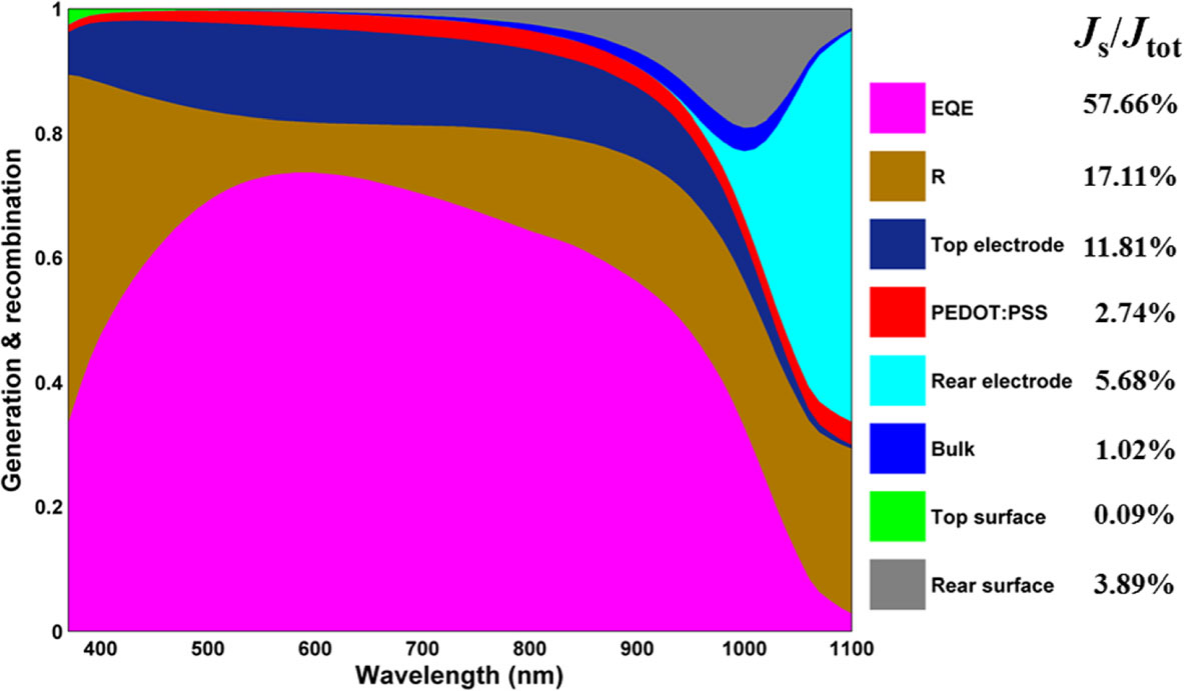
Optical generation and recombination inside the HHSCs for each part

First of all, the simulated reflection (*R*) and *EQE* spectra were compared with the experimental results. As shown in Fig. 1c, d, theoretical curves showed wonderful agreements with the experimental results over almost the entire spectra. As we focused on the reflection spectra in Fig. 1c, obviously, the reflection curves revealed standard monolayer anti-reflection (AR) nature (i.e., reflection values first decrease, and then increase, leaving the minimum value at *λ* = 600 nm). This is because the PEDOT:PSS with the refractive index (*n*) of about 1.2~1.6 matches with that of Si substrate. The best response wavelength (*λ* = 600 nm) is dependent on *n* as well as the thickness of the PEDOT:PSS layer [25]. The EQE of HHSCs that relies on the optical absorption of Si layer and carrier loss in electrical process was drawn in Fig. 1d. The photoelectrical loss will be discussed thoroughly in the next section. The short current density (*J*_sc_) that represents the integrated quantum efficiency is calculated by integrating the EQE spectrum of the cell under the standard AM1.5G illumination [26].

1 $$ {J}_{\mathrm{sc}}={\displaystyle {\int}_{300\mathrm{nm}}^{1200\mathrm{nm}}\frac{q\lambda }{hc}}{\varPhi}_{\mathrm{AM}1.5}\left(\lambda \right)\mathrm{E}\mathrm{Q}\mathrm{E}\left(\lambda \right)d\lambda, $$

where *q* is the unit charge, *h* is the Plank’s constant, *c* is the speed of light in vacuum, and *Φ*_AM1.5_ is the solar spectral irradiance under air mass 1.5G [27]. Similarly, other current densities that appeared in Fig. 2 were obtained by the same formula.

To have a comprehensive understanding on the processes of optical generation and electrical recombination, we presented the spectra as well as the equivalent current ratio (*J*_s_/*J*_tot_) for each part of the solar cell in Fig. 2, where *J*_s_ and *J*_tot_ represent the branched and total current density, respectively. Except for the EQE and *R*, the shielding loss by top Ag electrodes (top electrodes) is evaluated by considering the effective coverage area. The losses caused by parasitic absorption of PEDOT:PSS as well as the transmission of the SCs were also considered. Here, it is worth pointing out that the simulated transmission is slightly higher than that of the actual one in the long waveband as one can observe from the EQE spectrum in Fig. 1d. The reason is that the rear surface of Si is rough (i.e., truncated inverted nanopyramid) in our experiment, which contributed to the reduction in the transmission of the HHSCs due to scattering effect. This leads to inconsistency to the simulation (5.68% current density loss) where a flat configuration was taken into account. In our experiment and simulation process, the effective illumination area that lies on the comb-like hard mask we used in the thermal evaporation process was only about 85%, yielding a current density loss ratio of the top electrode up to 11.81%. Reflection is dependent on the refractive indexes of PEDOT:PSS and Si, as well as the thickness of PEDOT:PSS. They contribute the most important part of the optical losses (about 17.11%). The parasitic absorption of PEDOT:PSS produced a loss in the current density ratio of about 2.74% over the entire spectral range. Besides, the current density ratios inherent to the recombination inside the bulk, near the top and rear surfaces are 1.02, 0.09, and 3.89%, respectively. What is more, since we assumed an ideal interface between Si and PEDOT:PSS, neglecting the influence of the interface states, the top surface recombination can almost be ignored because of strong electrical passivation.

The generation, transportation, and collection of carriers played a key role in the analysis of the recombination procedure inside HHSCs; therefore, a detailed electrical simulation and discussion on these items need to be carried out. The wavelength-dependent photocarrier generation rate *G*(*λ*) can be expressed as the following equation:

2 $$ G\left(\lambda \right)=\frac{\varepsilon "\left(\lambda \right)\mid E\left(\lambda \right){\mid}^2}{2\hslash }{\varPhi}_{\mathrm{AM}1.5}\left(\lambda \right)d\lambda, $$

where *ε*″ is the imaginary part of the permittivity, *E* is the electric field, and ℏ is the reduced Planck’s constant. In this study, we assumed that the photon-generated carriers were completely ionized when suffering from a voltage barrier. Then, the separated carriers will transport across the HHSCs and collected by the extreme electrodes. Therefore, the effective collection efficiency (i.e., EQE) equals to the reduction of recombination in the internal area as well the interfaces in between the different materials from photocarrier generation, as shown in Eq. (4).

3 $$ \mathrm{E}\mathrm{Q}\mathrm{E}\left(\lambda \right)={j}_{\mathrm{s}}\ \left(\lambda \right)/q{b}_{\mathrm{s}}\left(\lambda \right) $$

4 $$ \begin{array}{l}{j}_{\mathrm{s}}\left(\lambda \right)=\raisebox{1ex}{$q{\displaystyle {\displaystyle \iiint G\left(\lambda \right)\mathrm{d}V}}$}\!\left/ \!\raisebox{-1ex}{${\displaystyle {\displaystyle \iint dS}}$}\right.-\raisebox{1ex}{$q{\displaystyle {\displaystyle \iiint {U}_{\mathrm{bulk}}\left(\lambda \right)dV}}$}\!\left/ \!\raisebox{-1ex}{${\displaystyle {\displaystyle \iint dS}}$}\right.-\raisebox{1ex}{${\displaystyle \iint {J}_{\mathrm{s}\mathrm{urf}}\left(\lambda \right)dS}$}\!\left/ \!\raisebox{-1ex}{${\displaystyle {\displaystyle \iint dS}}$}\right.,\\ {}\end{array} $$

where *j*_s_ the frequency-dependent photocurrent density coming from the effective carrier, *b*_s_ is the solar incident photon flux spectrum (AM1.5G), *U*_bulk_ and *U*_surf_ represent the recombination rate in the internal and surface, respectively, and *V* and *S* are the volume of the Si layer and surface area of the cell. For *U*_bulk_, three typical recombination that includes Shockley-Read-Hall (SRH), radiative (Rad), and Auger (Aug) recombination are considered [28–31].

5 $$ {U}_{\mathrm{bulk}}\left(\lambda \right)={U}_{\mathrm{SRH}}+{U}_{\mathrm{Aug}}+{U}_{\mathrm{Rad}}, $$

6 $$ {U}_{\mathrm{SRH}}=\frac{np-{n}_{\mathrm{i}}^2}{\tau_{\mathrm{n}}\left(p+{n}_{\mathrm{i}}\right)+{\tau}_{\mathrm{p}}\left(n+{n}_{\mathrm{i}}\right)}, $$

7 $$ {U}_{\mathrm{Aug}}=\left({C}_{\mathrm{n}}n+{C}_{\mathrm{p}}p\right)\left(np-{n}_{\mathrm{i}}^2\right), $$

8 $$ {U}_{\mathrm{Rad}}={B}_{\mathrm{rad}}\left(np-{n}_{\mathrm{i}}^2\right), $$

where *n* (*p*) is the electron (hole) concentration, *τ*_n_ (*τ*_p_) is the electron (hole) lifetime, *n*_i_ is the intrinsic carrier concentration, *B*_rad_ is the coefficient of bimolecular radiative recombination, and *C*_n_ (*C*_p_) the electron (hole) Auger coefficient. For temperature (*T*) = 300 K, *B*_rad_, *C*_n_, and *C*_p_ of Si are 9.5 × 10^−15^ cm^3^/s, 2.8 × 10^−31^ cm^6^/s, and 9.9 × 10^−32^ cm^6^/s, respectively. The electrical parameters of PEDOT:PSS were defined according to reference [32]. Surface recombination (*J*_surf_) was numerically modeled by the current density loss:

9 $$ {J}_{\mathrm{surf}}=q\updelta p{S}_{\mathrm{surf}}, $$

where δ*p* is the excess minority carrier concentration at the surface and *S*_surf_ is the surface recombination velocity.

**Fig. 3:**
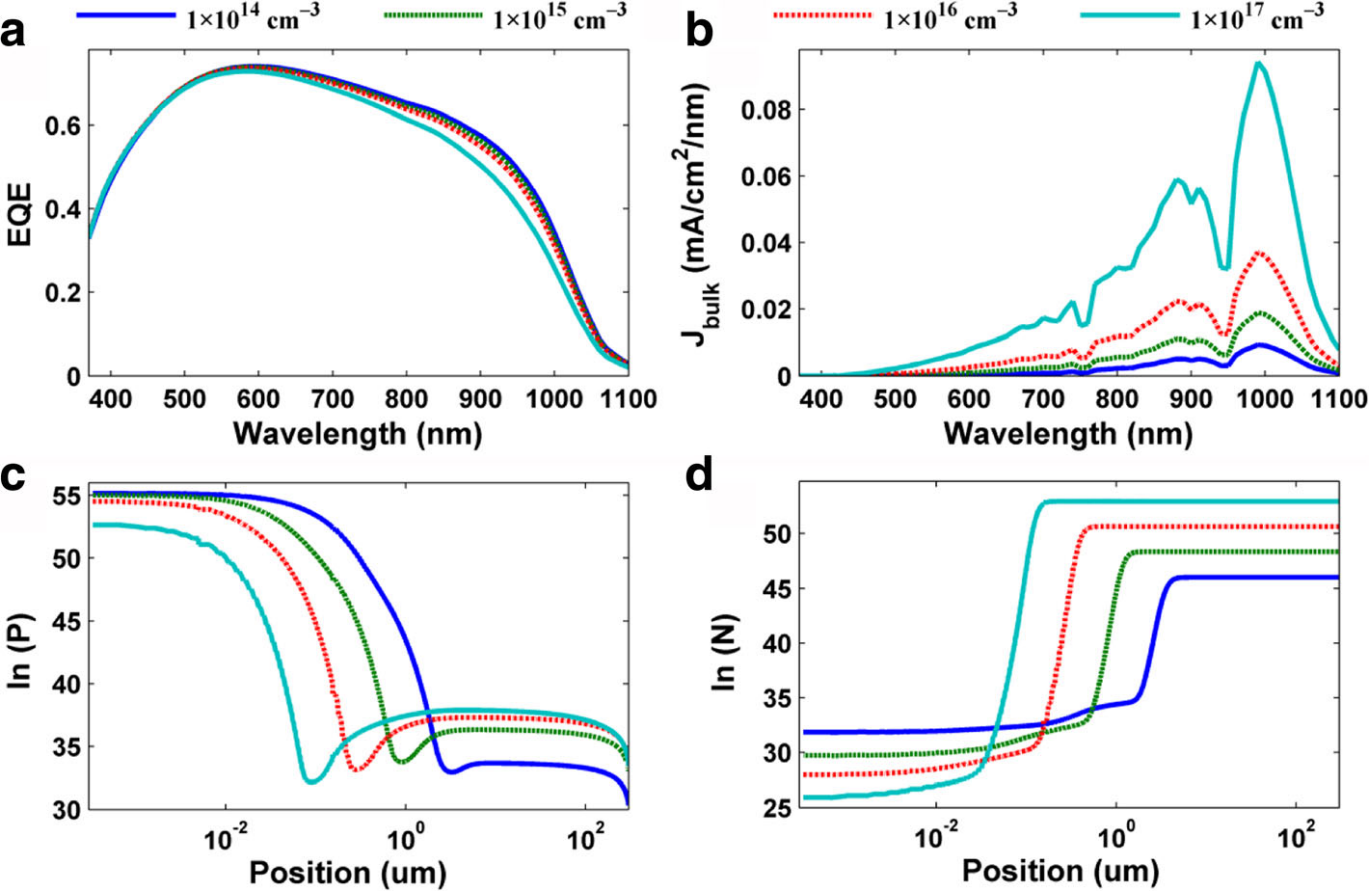
**a** EQE spectra. **b** Photocurrent densities of bulk recombination spectra. The stabilized distributions of **c** hole and **d** electron concentrations at *λ* = 500 nm under different doping concentrations of the Si substrate

In order to perform a comprehensive device-oriented simulation, two classical parameters (i.e., surface recombination velocity (*S*_surf_) and doping concentration of Si substrate) that characterize the electrical response of the HHSCs were discussed in the next section. Figure 3a, b shows the EQE spectra and photocurrent density of the bulk recombination spectra under different doping concentrations of the Si substrate (i.e., 1 × 10^14^, 1 × 10^15^, 1 × 10^16^, and 1 × 10^17^ cm^–3^). Besides, for better analysis, the stabilized distributions of the hole and the electron concentrations at *λ* = 500 nm were also plotted in Fig. 3c, d. We can find that (1) the hole concentration in the front interface (near the Si surface) is comparable to or even exceeds than that of electrons, indicating that the holes and electrons in this region turn into the majority and minority carriers, respectively, revealing that an inversion layer forms near the PEDOT:PSS and Si contact surface as mentioned before and (2) with the increase of doping concentrations of Si substrates, the width of the depletion layer is shorten and the stabilized concentrations of majority/minority carriers (electron/hole) inside the Si substrate were increased, correspondingly.

In this simulation, to ensure a fair comparison, we keep the rear surface recombination velocities at a constant value (i.e., 3 × 10^4^ cm/s) when investigating the EQE response of HHSCs under different doping concentrations, so the bulk recombination dominates the electrical losses in the transport process of the carriers. From the EQE spectra in Fig. 3a, it is easy to see that with the doping concentrations’ increases, the EQEs show a declining trend at *λ* > 500 nm, while maintaining a steady state at *λ* < 500 nm. This is because when *λ* < 500 nm, the injection of the carriers that concentrate in the upper surface of the HHSCs can be separated effectively by the built-in potential, leading to negligible bulk recombination as shown in Fig. 3b. As *λ* > 500 nm, the continuing and vigorous bulk recombination resulting from a longer diffusion length is the main reason for the atrophied EQE. With the increase of doping concentrations, the bulk recombination increases sharply according to the following reasons: (1) the reduced bulk lifetime results in SRH recombination increasing synchronously and (2) the increased excess minority carrier concentration (i.e., δ*p*) leads to the increase in bulk recombination.

**Fig. 4:**
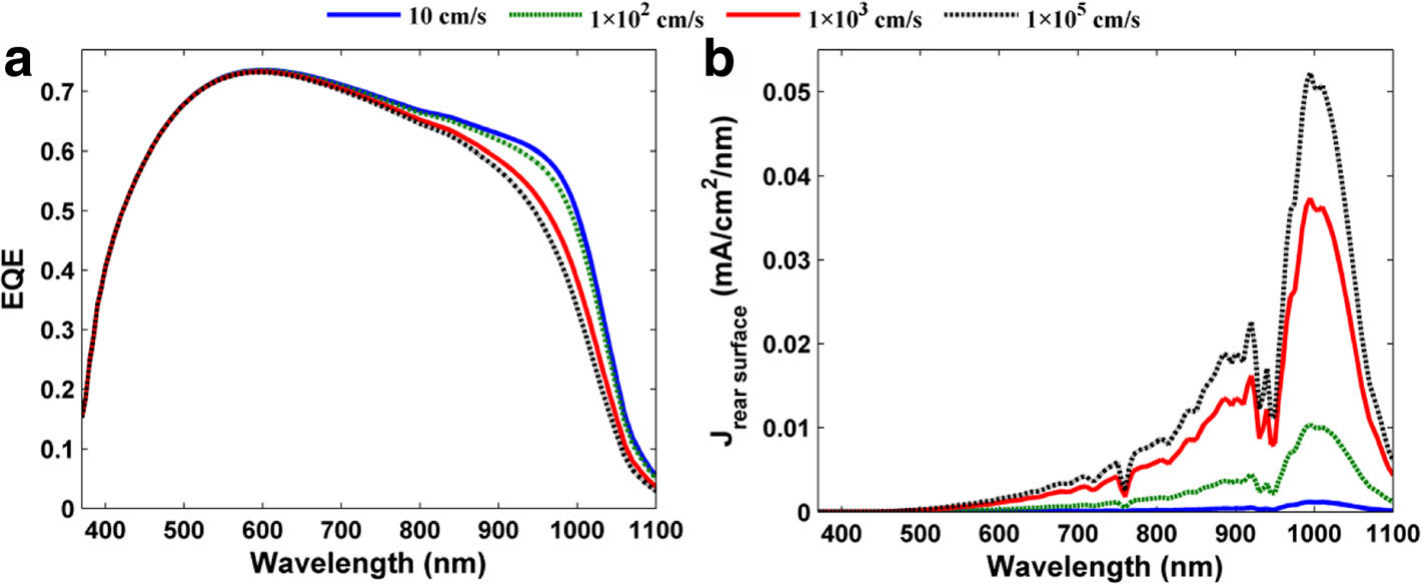
**a** EQE spectra and **b** photocurrent density spectra of the rear surface recombination under various surface recombination velocities

Finally, we briefly discussed the electrical performances of the HHSCs of various surface recombination velocities. Figure 4a, b revealed the EQE spectra and photocurrent density loss of the rear interface under four different surface recombination velocities (i.e., 1 × 10^1^, 1 × 10^2^, 1 × 10^3^, and 1 × 10^5^ cm/s), where the same doping concentration of the Si substrate was considered (i.e., 1.8 × 10^15^ cm^–3^). As shown in Fig. 4a, EQE decreases with increasing of *S*_surf_, especially at *λ* > 500 nm. This can be easily explained in this observation by the photocurrent density spectrum of the rear surface recombination as shown in Fig. 4b. For the given doping concentration of the Si substrate, the interface recombination dominates the electrical loss of the whole entire device, so the decays in EQEs are attributed to the booming recombination at interface.

## Conclusions

In summary, we have reported a comprehensively optoelectronic simulation on the PEDOT:PSS/Si hybrid heterojunction solar cells based on finite element method. By carefully addressing the electromagnetic and carrier-transport process, we predicted the current density losses, including the loss/recombination stemming from the reflection, top Ag electrode, parasitic absorption in the PEDOT:PSS and rear metal electrode, and the bulk and surface recombination. With the aid of the stabilized distributions of carrier concentration, the optoelectronic performance of HHSCs was fully discussed considering the influence of doping concentrations of Si substrate and surface recombination velocities. With increasing Si doping concentration and surface recombination velocities, the EQEs declined dramatically due to the increased excess minority carrier concentration or bulk recombination.

## Abbreviations

AR: Anti-reflection
EQE: External quantum efficiency
FEM: Finite element method
*G*: Photocarriers generation rate
HHSCs: Hybrid heterojunction solar cells
*J*_sc_: Short current density
*J*_surf_: Surface recombination current density
*k*: Extinction coefficient
*n*: Reflective index
PEDOT:PSS: Poly(3,4-ethylenedioxy thiophene):polystyrenesulfonate
PV: Photovoltaic
*R*: Reflection
SRH: Shockley-Read-Hall
*S*_surf_: Surface recombination velocity
